# Volatile, Sensory and Functional Properties of HydroSOS Pistachios

**DOI:** 10.3390/foods9020158

**Published:** 2020-02-06

**Authors:** Luis Noguera-Artiaga, Paola Sánchez-Bravo, David Pérez-López, Antoni Szumny, Ángel Calin-Sánchez, Armando Burgos-Hernández, Ángel A. Carbonell-Barrachina

**Affiliations:** 1 Department of Agro-Food Technology, Research Group “Food Quality and Safety”, Escuela Politécnica Superior de Orihuela (EPSO), Universidad Miguel Hernández de Elche (UMH), 03312 Orihuela, Alicante, Spain; lnoguera@umh.es (L.N.-A.); paola.sb94@gmail.com (P.S.-B.);; 2 GEIGRAM, Departamento de Producción Agraria, ETSIAAB, Universidad Politécnica de Madrid, 28040 Madrid, Spain; david.perezl@upm.es; 3 Department of Chemistry, Faculty of Biotechnology and Food Science, Wrocław University of Environmental and Life Sciences, 50-375 Wrocław, Poland; antoni.szumny@up.wroc.pl; 4 Departamento de Investigación y Posgrado en Alimentos, Universidad de Sonora, Sonora 83000, Mexico; armando.burgos@unison.mx

**Keywords:** antioxidant activity, fatty acid methyl esters, hydroSOS, *Pistacia vera*, pistachio flavor, quality, sensory analysis, total polyphenol content

## Abstract

Climate change, the increase in world population, and the intensification of urban and industrial activities, will cause a shortage of water for agriculture. This situation requires conscientious studies to manage water deficits without affecting the quality of the crops. In this study, regulated deficit irrigation (RDI) strategies and three rootstocks (*P. atlantica*, *P. integerrima*, and *P. terebinthus*) were applied to pistachio cultivation to study the quality of fruits obtained based on the morphological, functional, aroma, and their sensory properties. The results obtained demonstrated that RDI T1 (during phenological phase II of cultivation the stem water potential was maintained around −1.5 MPa) led to pistachios with same morphological properties, total polyphenol content, antioxidant activity, volatile composition, sensory properties, better profile of fatty acids, and being the favorite ones for international consumers, as compared to pistachios obtained under full irrigation treatments. On the other hand, when *P. integerrima* was used, pistachios obtained had the highest weight, the lowest content of sucrose and the best functional properties.

## 1. Introduction

Mediterranean and South American countries, Southern California, Southern Australia and South Africa are characterized by partially wet springs and autumns, mostly rainy winters and hot dry summers. Water scarcity and water deficits in plants, mainly due to scarce rainfall, must be supplemented with irrigation treatments. In addition, different factors, such as climate change, the increase in world population, and the intensification of urban and industrial activities, will cause a shortage of water for agriculture, and it will become more and more severe in the near future [1]. This situation requires more conscientious studies to manage water deficits without affecting the quality of crops. These studies should focus on crops which are able to withstand deficit irrigation or have low water needs but without drastic impacts on production and fruit quality [2].

One of the techniques focused on the reduction of irrigation water during fruits and vegetables farming is regulated deficit irrigation (RDI). RDI consists of the imposition of water deficits in specific phenological stages, which are less sensitive to water stress without affect the crop yield or its economic benefits [3,4].

Pistachio (*Pistacia vera*) is considered the only commercially edible nut among the different species in the genus *Pistacia*, and it has been cultivated for centuries in Mediterranean areas and is considered resistant to both drought and salinity [5]. This tolerance is based mainly on crop yields, but the physicochemical, functional and sensory quality of nuts has not been fully characterized. For their vegetative propagation, pistachio trees requires the use of rootstocks, because they cannot be propagated by cutting and planting because this propagation material do not produce enough roots [6]. The main rootstocks used for pistachio cultivation are *P. atlantica* Desf., *P. integerrima* L., *P. terebinthus* L. and *P. vera* L. [7]. Cultivation of pistachio trees has become a very profitable business, because in recent years, their harvesting was fully mechanized, the inputs associated to their cultivation has decreased, and the prize paid to producers is constantly increased [2]. In the future, this trend is expected to keep increasing due to the many studies supporting the health benefits observed after pistachio consumption [8,9]. It has been proved that the pistachio antioxidant capacity, total phenolic content, monounsaturated and polyunsaturated acids, lutein, phytosterols, and another functional compounds (founded on the pistachio nuts) were responsible for the anti-inflammatory potential, helping to promote cardiovascular health, and foster protective effect against colorectal and breast cancer of this nuts [10,11,12,13].

For all the above reasons, it is necessary to establish or identify those parameters that allow characterizing the quality of pistachios. In this sense, the main objective of this study was to evaluate the quality of pistachio nuts obtained using three irrigation treatments and three rootstocks, based on their morphological properties, fatty acids content, antioxidant properties, total polyphenol content, volatile composition and their sensory properties.

## 2. Materials and Methods

### 2.1. Plant Material, Growing Conditions and Experimental Design

Pistachio nuts from trees (*P. vera*), cultivar “Kerman” were collected during 2016 from the experimental orchard “La Entresierra” located at Ciudad Real, Spain (3°56’ W, 39° N; altitude 640 m above sea level). This area is characterized by a Mediterranean climate, with an average annual rainfall of 397 mm. The soil is a shallow clay-loam (Petrocalcic Palexeralfs) with a discontinuous petrocalcic horizon located at 0.5 m with a pH about 8.1, low electrical conductivity (0.2 dS/m), 1.05% organic matter, 0.12% N, 17 × 10^−4^ mol/kg K and a high cation exchange capacity (0.186 mol/kg).

Eighteen plots were used for this study with a completely randomized factorial design. Each of these plots had 12 trees (2 on the center for the analyses and 10 surrounding them) with same conditions of irrigation and rootstock. Pistachio trees were grafted over 3 rootstocks: (i) *P. atlantica*, (ii) *P. integerrima* and (iii) *P. terebinthus*, and, 3 irrigation treatments: (i) T0, in which trees were irrigated to ensure non-limiting water conditions in the soil (100% ETC of the previous week); (ii) T1, in which irrigation was suppressed (during phase II) until pistachio trees reached a stem water potential (SWP) below −1.5 MPa; and (iii) T2 with same irrigation protocol as T1 but with a SWP threshold of −2.0 MPa. Water relations were evaluated according to Memmi [14].

Pistachio nuts were collected from the field, and after being peeled and dried (convection oven with hot air at 60 °C until a moisture content of 5%), were immediately vacuum-packed and posted to the Universidad Miguel Hernández de Elche facilities in Orihuela (Alicante, Spain). Once there, samples were kept at 4–5 °C until analysis.

### 2.2. Volatile Compounds

The extraction of the volatile compounds of the samples of pistachios was carried out using the headspace solid-phase micro-extraction (HS-SPME) method. A sample of 1 g of ground pistachios was placed on a 50 mL vial, with a magnetic bar, and closed with an aluminum layer (foil). After equilibration time, 5 min at 45 °C, a 30/50 μm fiber (SUPELCO) covered by DVB/CAR/PDMS (Divinylbenzene/Carboxen/Polydimethylsiloxane) was exposed to the vial headspace at 45 °C, with continuous agitation (500 rpm) in a magnetic stirrer (IKA C-MAG HS 4, IKA-Werke GmbH & Co. KG, Staufen, Germany). After 25 min of exposure, fiber was put in a gas chromatograph to analyse.

The isolation and identification of the volatile compounds previously extracted by HS-SPME were performed using a Saturn 2000 Varian Chrompack gas chromatograph (Varian, Inc., Palo Alto, CA, USA) with an HP-5 column (5% Phenyl Methylpolysiloxane) 30 m × 0.53 mm ID × 1.0 µm (Agilent, Santa Clara, CA, USA). A mass spectrometer equipped with an ion-analyzer was set to 1508 V for all analyzes, and an electronic voltage factor to 1350 V. The analysis was carried out from 39 to 400 m/z, with an electronic impact (EI) of 70 eV, in 1 scan/s mode. Helium was used as carrier gas at a flow rate 1.0 mL/min and with a split ratio of 1:20. The injector and detector temperatures were 200 and 300 °C, respectively. The oven temperature started at 40 °C and, after 3 min, was increased by 5 °C/min up to 110 °C. Then, the temperature was increased by 20 °C/min up to 270 °C. The total analysis program lasted 25 min.

The volatile compounds were identified using three analytical methods: (i) Kovats Index (KI), (ii) GC-MS retention index (original chemical compound), and (iii) mass spectrum (original chemical compound and collection of the NIST05 and Adams 2012 spectrum library). The retention indexes were calculated using standard of aliphatic hydrocarbons in the range from C–5 to C–23. For the identification and determination of volatile compounds, the MS Workstation (Version 6.5, 2005 Varian, Palo Alto, CA, USA) and MestReNova (Version 9.0.1, 2014, Mestrelab Research, Santiago de Compostela, Spain) programs were used.

### 2.3. Sensory Analysis

The sensory analysis of samples was focused only on the analysis of the pistachios obtained under different irrigation treatments, in order to minimize the number of samples and, thus, maintain the panelists concentration to the maximum. Based on previous results [7], pistachio nuts obtained by *P. atlantica* rootstock were used on the sensory study.

To obtain information about the consumer opinion on the sensory properties of pistachios, a sensory evaluation with consumer panel was carry out in 3 countries: Mexico, Poland, and Spain. At least 60 consumers were recruited in each country. Consumers had to complete a screening questionnaire stating their age, gender, and allergies or diet restrictions. Consumers were asked about nut consumption frequency and willingness to taste pistachios. Consumers who stated that they were 18–70 years old, had no diet restrictions or allergies, ate any kind of nut at least once per week and were willing to taste pistachios were recruited for testing. In the specific case of Poland, the ballots, screeners and demographic questionnaires were translated from Spanish to Polish, and, then, back to Spanish.

Ten pistachio nuts were served, to each panelist, in odor-free disposable 60 mL covered plastic cups, coded using three-digit numbers, and at room temperature. Unsalted crackers and drinking water were used between samples to clean the panelists’ palate. Natural illumination was used during the test, and testing room was at 20 ± 2 °C.

Consumers responded using a 9-point hedonic scale, where 9 = like extremely and 1 = dislike extremely. Consumers were, then, asked to indicate their order of preference for the samples, and mark the reasons of their preference regarding the attributes under study (size, peel, color, pistachio-ID, toasted, sweet, sour, aftertaste, oiliness, hardness, crunchiness, friability and adhesiveness). Then, consumers were asked about their “global” satisfaction degree for the samples under evaluation and for their intent to purchase.

### 2.4. Determination of Sugars and Organic Acids

Sugars and organic acids were identified and quantified according to Hernández [15], with some modifications. Approximately 1 g of sample was diluted in 5 mL of phosphate buffer (pH 7.8), homogenized by Ultra-TurraxTM (IKA L004640, Staufen, Germany) for 1 min, and centrifuged at 15,000× *g* for 10 min. Finally, samples were filtered through a 0.45 μm Millipore filter. For the determination of the content of sugars and organic acids on samples, an HPLC (high-performance liquid chromatograph) Hewlett-Packard series 1100 (Hewlett-Packard, Wilmington, DE, USA) was used. The elution buffer consisted of 0.1% phosphoric acid with a flow rate of 0.5 mL/min.

Sugars and organic acids were isolated using a Supelco column (Supelcogel TM C-610H column 30 cm × 7.8 mm, Supelco, Inc., Bellefonte, PA, USA) and a precolumn Supelguard (5 cm × 4.6 mm; Supelco), and the absorbance was measured at 210 nm using a diode-array detector (DAD). Standards of sugars (glucose, fructose, sucrose, raffinose, maltitol, and sorbitol) and organic acids (oxalic, citric, tartaric, malic, quinic, shikimic, succinic and fumaric) were obtained from Sigma (Poole, UK). Calibration curves were used for the quantification of sugars and organic acids, showing good linearity (*R*^2^ = 0.999). Results for both organic acids and sugars were expressed as concentrations g/L of dry weight (dw).

### 2.5. Total Polyphenol Content and Antioxidant Activity

For the total polyphenol content (TPC) determination and the antioxidant activity of the pistachios affected by rootstock and irrigation treatments, a methanol extract was prepared. Half a gram of pistachios (crushed with a grounder) was introduced in a test tube with 10 mL of MeOH/water (80:20, *v*/*v*) in 1% HCl. Then, the mixture was sonicated at 20 °C for 15 min and left at 4 °C for 24 h. After that, the extract was sonicated again for 15 min and centrifuged at 10,000× *g* for 10 min [16].

TPC was quantified using the Folin-Ciocalteu colorimetric method described by Gao [17], with some modifications. To 0.1 mL of the methanolic extract was added 2 mL of distilled water, 0.2 mL of Folin-Ciocalteu reagent, and was incubated for 3 min at room temperature. After that, 1 mL of 20% sodium carbonate was added and the mixture was incubated again for 1 h [16]. The absorbance was determined by measurement at 765 nm using an UV-visible spectrophotometer (Thermo Fisher Scientific Helios Gamma model, UVG 1002E, Waltham, MA, USA). Quantification was carried out according to the standard curve of gallic acid. The results were expressed as gallic acid equivalents (GAE), mg/kg (dw).

For the analysis of the antioxidant activity, the methods ABTS^+^ [18], FRAP [19], and DPPH^•^ [20] were used. Ten microliters of the supernatant of the methanolic extract was mixed with 990 μL of reagent ABTS^+^ or FRAP. After a reaction time of 10 min, the absorbance was measured at 734 nm for ABTS^+^ and 593 nm in case of FRAP method. For the DPPH method, 10 μL of the supernatant was mixed with 40 μL of MeOH and 950 μL of DPPH^•^ solution. Then, the mixture was shaken, placed under dark conditions (15 min), and its absorbance was determined at 515 nm. The results obtained on the analysis of the antioxidant activity of pistachio samples were expressed as mmol Trolox/kg dw.

### 2.6. Fatty Acids

The determination of fatty acid methyl esters (FAMEs) was carried out according to Noguera-Artiaga [16]. A gas chromatograph Shimadzu GC-17A (Shimadzu Corporation, Kyoto, Japan), coupled with a Shimadzu mass spectrometer detector (GCMS QP-5050A) was used for the analysis of the organic layer of the pistachio extracts. The chromatograph was equipped with a column (polar) Suprawax 280, 100% polyethylene glycol (Teknokroma S. Co. Ltd., Barcelona, Spain; 30 m × 0.25 m × 0.25 µm film thickness). Helium (flow rate of 1.1 mL/min) was used as a carrier gas (split ratio 1:10). The temperature on the injector was 230 °C; on the detector, the temperature was 260 °C. The oven program and the identification of peaks were carry out following the method described by Carbonell-Barrachina [7] and Noguera-Artiaga [16].

### 2.7. Morphological Analysis

Twenty-five pistachio nuts from each treatment were randomly selected and used to determine the size, weight, and color. In addition, pistachios were classified as non-split open, split open, and others (uncommercial: unpeeled, broken shell, dark color, etc.). For the determination of the size, the height, width and length of the edible part from each pistachio were measured using a digital caliper (model 500-197-20 150 mm; Mitutoyo Corp., Aurora, IL, USA). In case of weight, the whole nut, shell and edible part were weighed (model AG204 scale; Mettler Toledo, Barcelona, Spain) with a precision of 0.1 mg. For the color, these 25 pistachios were ground (Taurus Aromatic Ver II; Taurus Group, Barcelona, Spain) and placed in Petri dishes (100 mm × 15 mm). A colorimeter Minolta model CR-300 (Minolta, Osaka, Japan) with an illuminant D65 and an observer of 10° was used for the measuring of color of samples. Color data were provided as CIEL*a*b* coordinates.

### 2.8. Statistical Analysis

The data presented in this study are the mean values of 3 replicates and was subjected to two-way (irrigation treatment and rootstock) analysis of variance (ANOVA). Then, data were subjected to Tukey’s multiple-range test to compare the means. Percentage data were transformed by Arcosen function before statistical analysis. Differences were considered statistically significant at *p* < 0.05. All statistical analyses were done using XLSTAT software (Addinsoft, version 2014.1, Paris, France).

## 3. Results and Discussion

### 3.1. Volatile Compounds

Thirty-one compounds were identified in the volatile profile of pistachios under study (Table S1) and were characterized according their sensory descriptors. The three most abundant compounds were α-pinene (~35%), limonene (~14%), and β-myrcene (~11%), and it was demonstrated that both irrigation and rootstocks significantly affected the content these compounds (Table 1). These results agreed with those obtained by Hojjati [21] and Penci [22] in previous studies, who also found that the same main compounds predominated in the aromatic profile of pistachios and its essential oils, respectively.

The volatile composition of the T1 pistachios showed no significant differences with that of the control samples (T0). On the other hand, pistachios obtained under RDI T2 had lower amounts of α-pinene, dodecane and tridecane, but higher ones of β-myrcene and limonene than T0. Regarding the effect of the pistachio rootstock, *P. integerrima* had the highest content of α-pinene (the predominant compound); *P. atlantica* led to nuts with the highest content of β-myrcene, dodecane and tridecane; and, *P. terebinthus* had the highest content of limonene.

In previous studies, Carbonell-Barrachina [7] demonstrated that RDI treatments led to pistachio nuts with similar or even higher amounts of the main volatile compounds. These results are in concordance with those obtained in the current study, in the sense that both studies demonstrated that the application of RDI strategies had no negative effects on the volatile composition of pistachio nuts.

Based on the study of the interaction between the two studied factors (irrigation and rootstock), to obtain pistachios with the highest possible content of α-pinene, it is necessary to use the rootstock *P. integerrima* and the irrigation treatments T0 or T1. On the other hand, if the objective is to obtain pistachios with more citrus aroma (more limonene), the combination of *P. terebinthus* rootstock and irrigation treatment T1 will be the most successful one.

According to results obtained, the main volatile compounds found in the volatile profile of pistachios α-pinene, limonene, and β-myrcene (compounds sensory related with descriptors of woody, citrus and fruity, respectively) can be used as indicators to evaluate the pistachio aroma quality.

### 3.2. Sensory Analysis

Around 200 consumers from Mexico, Spain and Poland (at least 60 in each country) participated in the pistachio affective sensory analysis. In Mexico, a 68% of panelist were women, 37% in Poland, and 55% in case of Spain. Of the total number of consumers, 35% were between 18 and 25 years old, 32% were between 26 and 35, 15 % between 36 and 45 years old, 17% between 46 and 55 years old, and 2% were older than 55 years old.

The irrigation treatments significantly affected three out of the 13 sensory attributes under analysis (Table 2): pistachio-ID, oiliness, and overall. Pistachios obtained under RDI T1 obtained higher intensities of pistachio-ID (6.7) than control and T2 (6.4 and 6.4). This result was observed in each of the countries under study (Table 2).

The oiliness of T1 and T0 samples were slightly but statistically higher, 6.0 than T2 samples, 5.8. Mexican consumers liked the oiliness of the pistachio samples less than Polish and Spanish consumers; a similar trend was observed for hardness and crunchiness. The most consumed dried fruit in Mexico is peanut, so it is possible that consumers in this country are used to high intensities of these attributes and were expecting a little more oiliness, hardness and crunchiness in pistachio samples, hoping to find a texture similar to that of fried peanuts.

In addition, in case of overall liking (the attribute that define the final opinion of consumers about the overall quality of sample), the T1 treatment obtained the highest score (6.7), while T0 got 6.3; T2 was statistically related with both of them (score of 6.5). Regarding the factor country, this same trend was observed in Mexico and Spain. However, there were no statistically significant differences in the satisfaction degree of Polish consumers regarding the three irrigations treatments (Table 2).

**Figure 1:**
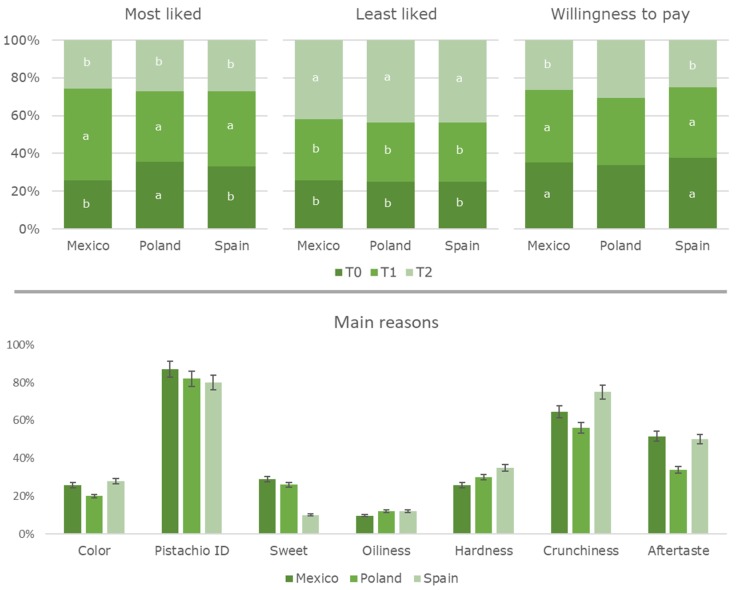
Preference of Mexican, Polish, and Spanish consumers about pistachios obtained using different irrigation treatments (T0, control; T1, moderate RDI regulated deficit irrigation); T2, severe RDI), together with their willingness to pay and the main reasons behind their election. Factors with the same letter were not significantly different (*p* < 0.05), Tukey’s least significant difference test.

When consumers were forced to choose (among the three samples studied) which was their favorite sample, T1 pistachios were the most liked ones in each of the countries (in case of Poland, no statistically significant differences were observed between T1 and T0). On the other hand, the least liked sample in all countries was T2 (Figure 1). Similar trends were observed when consumers were asked about their willingness to pay for the samples under study. Consumers mentioned that the main reasons for selecting the preferred sample (the most liked one) were: (i) pistachio flavor (~83%), (ii) crunchiness (~65%), (iii) aftertaste (~45%) and (iv) hardness (~30%) (Figure 1). Similar results were reported in previous studies; for instance, Carbonell-Barrachina [7] studied the purchase drivers of international pistachio consumers were pistachio flavor, saltiness, crunchiness and toasted flavor. Although, Noguera-Artiaga [23] concluded that international consumers preferred intense crunchy but low salty nuts.

### 3.3. Sugars and Organic Acids

Three sugars (maltitol, raffinose and sucrose) and three organic acids (fumaric, oxalic, and shikimic) were identified and quantified in the pistachio samples under study (Table 3). In a previous study, Luh [24], identified four main sugars present in pistachios: sucrose, fructose, glucose and raffinose, being sucrose the main sugar, representing ~40% of the total content.

T1 samples had lower concentration of fumaric acid (0.287 g/L) than control pistachios (T0 = 0.315 g/L), while T2 nuts (0.287 g/L) were statistically related with both T0 and T1. In the rest of organic acids and sugars, no statistically differences were found among pistachios obtained under different irrigation treatments (Table 3).

Regarding rootstocks, *P. integerrima* led to pistachios with lower sucrose concentration (19.77 g/L) than *P. terebinthus* and *P. atlantica* (22.51 and 24.98 g/L, respectively). In the analysis of organic acids, *P. integerrima* was the rootstock having the highest concentrations of the three studied acids, while *P. terebinthus* had the lowest amount of oxalic and fumaric acids (Table 3).

According to interaction of the two factors studied, pistachios obtained under *P. integerrima* and irrigation T1 had the lowest concentration of sucrose, while rootstock *P. atlantica* and T2 led to pistachios with the highest concentration of this sugar (Table 3).

Similar results were obtained by Lipan [25] in almonds affected by different treatments of regulated deficit irrigation (with sucrose, the predominant sugar showing no differences due to irrigation treatments).

According to our acknowledgement, these are the first results published regarding the effect of deficit irrigation on the acid and sugar composition of pistachios.

### 3.4. Antioxidant Activity (AA) and Total Polyphenol Content (TPC)

Results obtained on the study of AA and TPC are shown in Table 4. In general, pistachios have high functional potential based on their high total polyphenol content (~1350 mg GAE/kg, dw) and their antioxidant activity (~22 mmol Trolox/kg, dw, on the three methods studied). Similar results of TPC were obtained by Hojjati [21] in roasted pistachios, Lipan [25] in almonds, and Suárez [26] in chestnut.

The application of moderate regulated irrigation treatments (T1) on the cultivation of pistachios had no statistically incidence on the AA and TPC of nuts. On the contrary, when the water restriction was severe (T2), the AA of pistachios was reduced (according to DPPH and FRAP methods). Under situations of moderate water stress, plants redistribute the CO_2_ to the formation of secondary metabolites as a physiological response for the removal the free radicals formed; while under high stress, this CO_2_ is mainly used by primary metabolism [27,28]. Same results were found, in previous studies under similar conditions, by Noguera-Artiaga [16] who obtained that hydroSOS pistachios had same or even higher TPC than control samples. Similar results of AA and TPC were found in previous studies with pistachios affected by different irrigation treatments [4,7,29].

In case of the study of rootstocks, *P. integerrima* led to obtain pistachio nuts with the highest concentrations of TPC and AA than rootstocks *P. atlantica* and *P. terebinthus* (Table 4). In previous studies, no significant differences were found in the functional composition of pistachios obtained through these same rootstocks.

### 3.5. Fatty Acids

Nine fatty acids (FAMEs) were identified by GC-MS in pistachio samples (Table 5): two were polyunsaturated (PUFAs) [α-linolenic acid (C18:3) and linoleic acid (C18:2)]; three monounsaturated (MUFAs) [eicosenoic acid (C20:1), oleic acid (C18:1), and palmitoleic acid (C16:1)]; and, four saturated (SFAs) [arachidic acid (C20:0), stearic acid (C18:0), palmitic acid (C16:0), and myristic acid (C14:0)]. The three predominant compounds were C18:1 (~53% of the total), C18:2 (~31%), and C16:0 (~12%).

Pistachios obtained under moderate RDI (T1) had the highest content of oleic acid and the lowest one of α-linolenic as compared to those of control (T0) and T2 treatment (Table 5). In case of rootstocks, no statistically differences were observed on the fatty acid composition except on the content of α-linolenic acid, where *P. atlantica* had the lowest values (Table 5). Regarding the interaction between the two factors studied, rootstock and irrigation, pistachios obtained by *P. integerrima* and T1 had the highest content of oleic acid (Table 5).

**Figure 2:**
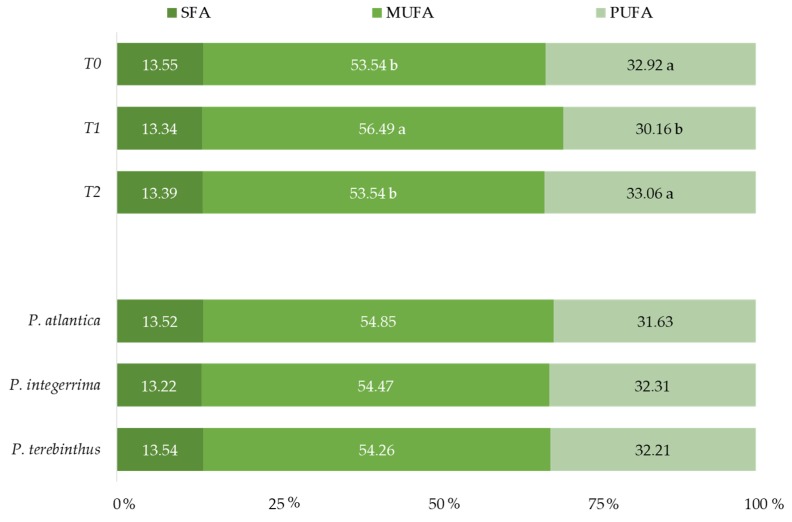
Fatty acid composition of pistachios as affected by deficit irrigation treatment and rootstock, grouped according to their saturation. Values (mean of 3 replications) followed by the same letter, within the same factor, were not significantly different (*p* < 0.05), Tukey’s least significant difference test. SFAs = saturated fatty acids; MUFAs = monounsaturated fatty acids; PUFAs = polyunsaturated fatty acids.

The application of treatment T1 affected the unsaturated fatty acid composition of pistachios, increasing the content of MUFAs, and decreasing that of PUFAs. The use of different rootstocks had no significant effect on the composition of the SFAs, MUFAs or PUFAs (Figure 2).

In previous studies, carried out under similar conditions, Carbonell-Barrachina [7] obtained that moderate RDI increased the content of linoleic acid, while in this study the one that has been increased was that of the oleic acid. Acar [30] reported that main fatty acids found in pistachio were oleic, linoleic and palmitic acids as has been shown in the current study.

### 3.6. Morphological Analysis

On the analysis of split-open and non-split open pistachios (Table 6) no statistically significant differences were observed among samples obtained under different irrigation treatments, being the mean values 54% and 42%, respectively. In case of effect of rootstock, *P. terebinthus* had the highest number of split pistachios (60%) and, consequently, the lowest number of non-split open pistachios (35%); while data on *P. atlantica* and *P. integerrima* were statistically equivalent.

The moderated reduction of water during the phenological phase II of pistachios (T1) had no effect on the weight and size of the commercial nuts (Table 6). On the other hand, high reduction of water during this same phase (T2), led to pistachios with the lowest weight of their edible nut (T0 = 0.692 g and T2 = 0.673 g).

Regarding rootstocks, no statistically significant differences were found on the weight of whole nut and shell, and on the length and height of pistachio nuts. However, samples of *P. integerrima* and *P. atlantica* had the highest weight of the edible nut (Table 6).

The color of the samples had statistically significant differences in the parameters L* and a*, in case of irrigation and rootstock. These differences were minimum, and some authors have concluded that differences smaller than two units, as it is the current case (Table 6), are imperceptible for the human eye [31,32].

Similar results were previously reported by Carbonell-Barrachina [7] and Noguera-Artiaga [16], who showed that neither rootstocks nor RDI treatments significantly affected the morphological parameters of pistachios.

## 4. Conclusions

The results obtained in this study demonstrated that the application of a moderate deficit irrigation during pistachio cultivation (T1) led to pistachios with same morphological properties, total polyphenol content, antioxidant activity, volatile composition and sensory properties than pistachios obtained using full irrigation (T0). Moreover, T1 led to pistachios with better profile of fatty acids and being the sample preferred by international consumers. On the contrary, when the RDI was severe (T2), pistachio nuts had the lowest antioxidant activity, the lowest total polyphenols content, and the least preferred samples by consumers. In case of pistachios obtained using different rootstocks, *P. integerrima* led to pistachio nuts with the highest weight, the lowest content of sucrose and better functional properties than *P. atlantica* and *P. terebinthus*. These results demonstrated that it is possible to save irrigation water in pistachio farming, with low environmental and economic cost, and leading to pistachio nuts with same or even better quality attributes.

## Figures and Tables

**Table 1:** Relative content (%) of volatile compounds on pistachios affected by regulated deficit irrigation and rootstocks.

Compound	ANOVA Test ^Ϯ^			Irrigation (%)			Rootstock (%)			Irrigation × Rootstock (%)								
	Irrigation	Rootstock	Irrigation × Rootstock	T0	T1	T2	AT	IN	TE	ATxT0	ATxT1	ATxT2	INxT0	INxT1	INxT2	TExT0	TExT1	TExT2
Acetic acid	NS	NS	NS	0.46	0.32	0.30	0.33	0.36	0.39	0.33	0.26	0.39	0.59	0.17	0.32	0.45	0.53	0.19
Ethyl acetate	NS	NS	NS	1.04	0.39	0.46	0.69	0.75	0.45	0.85	0.63	0.59	1.48	0.20	0.58	0.79	0.35	0.21
Pentanone	NS	NS	NS	0.25	0.20	0.17	0.19	0.21	0.22	0.20	0.18	0.19	0.33	0.13	0.16	0.21	0.28	0.18
1-Methyl-1H-pyrrole	NS	NS	NS	3.52	4.57	2.82	2.72	2.92	5.26	1.95	3.84	2.39	3.04	3.28	2.44	5.58	6.58	3.62
1-Pentanol	NS	NS	NS	0.92	0.55	0.43	0.67	0.47	0.76	0.90	0.72	0.39	0.67	0.27	0.45	1.18	0.65	0.46
(*Z*)-3-Octene	**	NS	**	0.26 b,^‡^	0.20 b	0.50 a	0.35	0.27	0.35	0.18 b	0.15 b	0.71 a	0.27 ab	0.13 b	0.39 ab	0.31 ab	0.33 ab	0.41 ab
Hexanal	NS	NS	NS	1.38	0.70	0.94	0.98	0.74	1.30	1.17	0.84	0.91	0.80	0.39	1.03	2.16	0.86	0.88
2-Octene	NS	NS	NS	0.18	0.20	0.27	0.22	0.18	0.24	0.17	0.17	0.31	0.15	0.15	0.24	0.22	0.27	0.24
1-Hexanol	NS	NS	NS	4.39	2.63	2.64	3.98	2.70	2.97	5.75	3.66	2.55	2.86	1.98	3.27	4.56	2.26	2.09
(*E*)-4-Nonene	NS	NS	**	0.21	0.21	0.30	0.27	0.20	0.24	0.18 b	0.20 ab	0.42 a	0.19 ab	0.14 b	0.26 ab	0.24 ab	0.27 ab	0.21 ab
(*Z*)-4-Nonene	NS	NS	NS	0.22	0.18	0.22	0.22	0.15	0.24	0.20	0.18	0.28	0.15	0.11	0.19	0.31	0.24	0.18
Nonane	NS	NS	NS	0.25	0.23	0.27	0.29	0.21	0.24	0.30	0.23	0.35	0.20	0.17	0.26	0.26	0.29	0.19
α-Pinene	**	**	**	36.90 a	35.08 a	33.54 b	30.19 b	42.41 a	32.92 b	31.29 c	33.13 c	26.15 d	49.27 a	46.25 a	31.71 c	30.14 c	25.86 d	42.76 b
2-Pentanol	NS	NS	NS	0.37	0.40	0.60	0.56	0.41	0.41	0.23	0.47	0.98	0.43	0.24	0.55	0.45	0.50	0.26
1-Decene	NS	NS	NS	0.83	0.40	0.47	0.75	0.36	0.60	1.10	0.53	0.62	0.47	0.30	0.31	0.94	0.38	0.47
Sabinene	NS	NS	NS	0.48	0.40	0.40	0.38	0.42	0.48	0.43	0.32	0.38	0.54	0.47	0.26	0.47	0.41	0.57
3-Decene	NS	NS	NS	0.62	0.35	0.49	0.51	0.34	0.61	0.44	0.47	0.61	0.41	0.16	0.43	1.00	0.41	0.42
β-Myrcene	**	**	NS	8.39 b	9.58 b	14.89 a	13.21 a	9.79 b	9.86 b	6.77	12.66	20.22	8.16	6.12	15.09	10.26	9.97	9.37
Decane	NS	NS	NS	2.07	2.53	2.74	2.92	2.26	2.16	2.13	3.27	3.34	2.09	1.96	2.73	1.99	2.35	2.14
3-Carene	NS	NS	NS	0.24	0.39	0.39	0.29	0.34	0.39	0.34	0.26	0.26	0.19	0.52	0.31	0.18	0.39	0.61
Limonene	***	**	**	12.19 b	13.72 b	15.01 a	11.19 b	12.24 b	17.49 a	14.87 c	8.11 de	10.60 d	7.91 e	12.73 cd	16.07 bc	13.80 c	20.32 a	18.36 b
(*E*)-3-Hexenol	NS	NS	NS	0.92	0.94	1.06	1.17	0.82	0.92	0.84	1.23	1.43	0.81	0.64	1.01	1.10	0.94	0.72
2-Octen-1-ol	NS	NS	NS	0.74	0.97	1.11	1.21	0.85	0.75	0.76	1.32	1.54	0.77	0.67	1.12	0.67	0.93	0.65
2-Methyl-decane	NS	NS	NS	0.68	0.45	0.49	0.68	0.46	0.48	0.83	0.60	0.61	0.48	0.31	0.58	0.72	0.45	0.26
Terpinolene	NS	NS	NS	0.66	0.95	0.76	0.81	0.84	0.72	1.06	0.79	0.57	0.40	1.43	0.70	0.52	0.62	1.02
Undecane	NS	NS	NS	8.59	9.40	8.77	9.53	8.77	8.46	9.87	7.73	11.00	7.67	9.17	9.47	8.22	11.32	5.84
2-Nonen-1-ol	NS	NS	NS	1.70	0.90	0.94	1.46	1.04	1.04	2.51	0.81	1.06	1.03	1.19	0.90	1.57	0.69	0.88
1-Nonanol	NS	NS	NS	1.54	1.20	1.01	1.72	0.90	1.13	2.34	1.68	1.13	0.75	1.02	0.94	1.55	0.90	0.95
Dodecane	**	**	**	6.25 ab	8.02 a	5.29 b	8.32 a	5.71 b	5.54 b	7.73 ab	10.51 a	6.71 ab	5.19 b	6.45 ab	5.46 ab	5.83 ab	7.09 ab	3.70 b
Decanal	NS	NS	NS	0.41	0.51	0.46	0.54	0.45	0.39	0.50	0.56	0.56	0.42	0.49	0.45	0.31	0.46	0.38
Tridecane	**	**	**	2.85 ab	3.45 a	2.26 b	3.66 a	2.44 b	2.47 b	3.75 ab	4.50 a	2.73 ab	2.26 ab	2.76 ab	2.28 ab	2.54 ab	3.09 ab	1.76 b

^Ϯ^ NS: not significant at *p* < 0.05; ** significant at *p* < 0.01. ^‡^ Values (mean of 3 replications) followed by the same letter, within the same volatile compound and factor, were not significantly different (*p* < 0.05), Tukey’s least significant difference test. *P. atlantica* (AT), *P. integerrima* (IN) and *P. terebinthus* (TE).

**Table 2:** Sensory affective test of hydroSOS pistachios conducted in three countries: Spain, Mexico, and Poland.

Factor	Liking													
	Size	Peel	Color	Pistachio ID	Toasted	Sweet	Sour	Aftertaste	Oiliness	Hardness	Crunchiness	Friability	Adhesiveness	Overall
ANOVA Test ^Ϯ^														
Irrigation	NS	NS	NS	**	NS	NS	NS	NS	*	NS	NS	NS	NS	**
Country	NS	NS	NS	NS	NS	NS	NS	NS	*	*	**	NS	NS	NS
Country × Irrigation	NS	NS	NS	*	NS	NS	NS	NS	*	NS	NS	NS	NS	*
Tukey’s Multiple Range Test ^‡^														
Irrigation														
T0	6.6	6.8	6.4	6.4 b	6.4	5.8	5.8	6.3	6.0 a	6.5	6.4	6.0	5.7	6.3 b
T1	6.7	6.9	6.5	6.7 a	6.3	6.0	5.6	6.1	6.0 a	6.6	6.4	6.0	5.7	6.7 a
T2	6.5	6.7	6.3	6.4 b	6.5	5.8	5.9	5.9	5.8 b	6.5	6.5	6.2	5.4	6.5 ab
Country														
Mexico	6.5	6.8	6.3	6.3	6.5	5.8	5.8	5.8	5.6 b	6.2 b	6.0 b	5.9	5.5	6.4
Poland	6.9	6.9	6.5	6.7	6.4	5.9	5.8	6.3	6.0 a	6.8 a	6.7 a	6.2	5.9	6.7
Spain	6.5	6.6	6.3	6.5	6.2	5.9	5.6	6.3	6.2 a	6.6 ab	6.7 a	6.1	5.6	6.5
Country × Irrigation														
Mexico × T0	6.7	6.9	6.4	6.2 c	6.4	5.9	6.0	6.1	5.6 b	6.1	6.1	5.7	5.5	6.0 b
Mexico × T1	6.5	6.9	6.4	6.6 ab	6.5	5.8	5.5	5.9	5.7 b	6.4	5.9	6.0	5.7	6.6 a
Mexico × T2	6.2	6.6	6.2	6.2 c	6.7	5.7	5.8	5.4	5.6 b	6.2	5.9	6.1	5.1	6.4 ab
Poland × T0	6.7	6.9	6.5	6.6 ab	6.5	5.7	5.8	6.2	6.2 a	6.8	6.5	6.2	5.9	6.9 a
Poland × T1	6.9	6.9	6.4	6.8 a	6.3	6.3	5.8	6.1	6.1 a	6.8	6.7	6.1	6.0	6.7 a
Poland × T2	6.9	6.9	6.7	6.7 a	6.5	5.8	6.0	6.4	5.8 ab	6.8	6.8	6.2	5.7	6.6 a
Spain × T0	6.4	6.6	6.3	6.4 b	6.2	5.8	5.5	6.5	6.3 a	6.7	6.5	6.0	5.8	6.1 b
Spain × T1	6.8	6.8	6.5	6.8 a	6.0	6.0	5.6	6.3	6.2 a	6.5	6.7	5.9	5.5	6.8 a
Spain × T2	6.3	6.5	6.1	6.3 bc	6.4	5.8	5.8	6.0	6.0 ab	6.5	6.9	6.3	5.4	6.5 ab

^Ϯ^ NS: not significant at *p* < 0.05; * and **: significant at *p* < 0.05 and 0.01, respectively. ^‡^ Values (mean of 200 replications) followed by the same letter, within the same sensory attribute and factor, were not significantly different (*p* < 0.05), Tukey’s least significant difference test.

**Table 3:** Sugars (g/L) and organic acids (g/L) on pistachios obtained under regulated deficit irrigation and different rootstocks.

Factor	Sugars (g/L dw)			Organic Acids (g/L dw)		
	Raffinose	Sucrose	Maltitol	Oxalic	Shikimic	Fumaric
ANOVA Test ^Ϯ^						
Irrigation	NS	NS	NS	NS	NS	***
Rootstock	NS	***	NS	***	***	***
Irrigation × Rootstock	NS	***	NS	NS	NS	***
Tukey’s Multiple Range Test ^‡^						
Irrigation						
T0	9.793	22.831	4.111	0.156	0.535	0.315 a
T1	9.077	20.449	4.848	0.127	0.581	0.287 b
T2	10.351	23.972	5.796	0.148	0.555	0.296 ab
Rootstock						
*P. atlantica*	10.345	24.977 a	5.772	0.165 a	0.467 b	0.260 b
*P. integerrima*	9.517	19.770 b	5.257	0.151 a	0.737 a	0.328 a
*P. terebinthus*	9.359	22.505 a	3.727	0.116 b	0.468 b	0.309 a
Irrigation × Rootstock						
T0 × *P. atlantica*	9.091	23.692 ab	4.441	0.189	0.456	0.273 bc
T1 × *P. atlantica*	8.963	21.574 ab	4.447	0.145	0.418	0.266 bc
T2 × *P. atlantica*	12.982	29.666 a	8.427	0.160	0.527	0.241 c
T0 × *P. integerrima*	10.418	20.981 ab	3.911	0.137	0.701	0.339 a
T1 × *P. integerrima*	9.237	18.240 b	6.503	0.140	0.832	0.299 ab
T2 × *P. integerrima*	8.895	20.090 ab	5.355	0.175	0.678	0.347 a
T0 × *P. terebinthus*	9.868	23.821 ab	3.982	0.141	0.448	0.332 a
T1 × *P. terebinthus*	9.032	21.534 ab	3.595	0.096	0.494	0.295 abc
T2 × *P. terebinthus*	9.176	22.160 ab	3.605	0.112	0.461	0.300 ab

^Ϯ^ NS: not significant at *p* < 0.05; ***, significant at *p* < 0.001. ^‡^ Values (mean of 3 replications) followed by the same letter, within the same column and factor, were not significantly different (*p* < 0.05), Tukey’s least significant difference test. dw: dry weight.

**Table 4:** Total polyphenol content, TPC [mg gallic acid equivalents (GAE)/kg dry weigh, dw] and antioxidant activity, AA (mmol Trolox/kg dw) of pistachios as affected by deficit irrigation treatment and rootstock.

Factor	TPC	DPPH	FRAP	ABTS
	(mg GAE/kg dw)	(mmol Trolox/kg dw)		
ANOVA Test ^Ϯ^				
Irrigation	***	***	***	NS
Rootstock	***	***	***	***
Irrigation × Rootstock	***	***	***	***
Tukey’s Multiple Range Test ^‡^				
Irrigation				
T0	1390 ab	21.70 a	23.89 a	23.5
T1	1409 a	20.50 ab	24.45 a	23.3
T2	1297 b	18.77 b	19.66 b	22.0
Rootstock				
*P. atlantica*	1310 b	19.02 b	20.08 b	21.53 b
*P. integerrima*	1522 a	22.09 a	25.47 a	28.08 a
*P. terebinthus*	1265 b	19.87 b	22.44 ab	19.07 c
Irrigation × Rootstock				
T0 × *P. atlantica*	1294 bcd	20.06 ab	22.07 abc	22.78 bc
T1 × *P. atlantica*	1450 abc	19.51 ab	21.80 abc	22.85 bc
T2 × *P. atlantica*	1184 d	17.48 b	16.37 c	18.95 c
T0 × *P. integerrima*	1615 a	24.28 a	26.57 ab	29.01 a
T1 × *P. integerrima*	1460 ab	22.53 ab	30.02 a	28.26 a
T2 × *P. integerrima*	1489 ab	19.46 ab	19.82 bc	26.96 ab
T0 × *P. terebinthus*	1260 bcd	20.76 ab	23.03 abc	18.56 c
T1 × *P. terebinthus*	1317 bcd	19.47 ab	21.51 abc	18.71 c
T2 × *P. terebinthus*	1216 cd	19.37 ab	22.77 abc	19.93 c

^Ϯ^ NS: not significant at *p* < 0.05; ***: significant at *p* < 0.001. ^‡^ Values (mean of 3 replications) followed by the same letter, within the same column and factor, were not significantly different (*p* < 0.05), Tukey’s least significant difference test.

**Table 5:** Fatty acid composition of pistachios as affected by deficit irrigation treatment and rootstock.

Factor	Fatty Acids (%)								
	C14:0	C16:0	C16:1	C18:0	C18:1	C18:2	C18:3	C20:0	C20:1
ANOVA Test ^Ϯ^									
Irrigation	NS	NS	NS	NS	**	NS	**	NS	NS
Rootstock	NS	NS	NS	NS	NS	NS	*	NS	NS
Irrigation×Rootstock	NS	NS	NS	NS	*	*	NS	NS	NS
Tukey’s Multiple Range Test ^‡^									
Irrigation									
T0	0.07	12.03	1.28	1.28	51.94 b	32.18	0.73 a	0.16	0.32
T1	0.07	11.60	1.21	1.52	54.95 a	29.54	0.62 b	0.16	0.33
T2	0.07	11.86	1.19	1.32	52.05 b	32.39	0.67 ab	0.15	0.30
Rootstock									
*P. atlantica*	0.07	11.90	1.20	1.38	53.34	31.00	0.63 b	0.18	0.31
*P. integerrima*	0.08	11.63	1.19	1.37	52.95	31.63	0.67 ab	0.15	0.33
*P. terebinthus*	0.07	11.96	1.29	1.36	52.66	31.48	0.73 a	0.15	0.31
Irrigation × Rootstock									
T0 × *P. atlantica*	0.07	12.13	1.26	1.28	52.09 b	32.01 ab	0.64	0.20	0.31
T1 × *P. atlantica*	0.07	11.59	1.17	1.29	52.95 b	31.87 ab	0.61	0.17	0.28
T2 × *P. atlantica*	0.06	11.97	1.17	1.58	54.98 ab	29.12 ab	0.63	0.16	0.33
T0 × *P. integerrima*	0.08	11.95	1.30	1.28	50.96 b	33.18 ab	0.78	0.14	0.33
T1 × *P. integerrima*	0.07	11.27	1.10	1.69	58.44 a	26.33 b	0.58	0.19	0.33
T2 × *P. integerrima*	0.08	11.66	1.18	1.16	49.45 b	35.38 a	0.66	0.11	0.33
T0 × *P. terebinthus*	0.07	12.01	1.28	1.28	52.76 b	31.36 ab	0.78	0.15	0.31
T1 × *P. terebinthus*	0.06	11.93	1.35	1.58	53.47 b	30.41 ab	0.68	0.14	0.37
T2 × *P. terebinthus*	0.08	11.93	1.23	1.23	51.73 b	32.65 ab	0.73	0.17	0.25

^Ϯ^ NS: not significant at *p* < 0.05; * and **, significant at *p* < 0.05 and 0.01, respectively. ^‡^ Values (mean of 3 replications) followed by the same letter, within the same column and factor, were not significantly different (*p* < 0.05), Tukey’s least significant difference test.

**Table 6:** Weight, size, and nature of pistachios as affected by deficit irrigation treatment and rootstock.

Factor	Weight (g)			Size (mm)			Number Per 100 Units				Color Coordinates				
	Whole	Edible Nut	Shell	Length	Height	Width	Split Open	Non-Split Open	Others ^§^	*L*		a*	b*	*Chroma*	Hue
ANOVA Test ^Ϯ^															
Irrigation	NS	***	NS	NS	***	***	NS	NS	NS	***		NS	NS	NS	***
Rootstock	NS	***	NS	NS	NS	***	***	***	NS	***		***	NS	NS	***
Irrigation × Rootstock	NS	***	NS	NS	***	***	*	*	NS	***		***	NS	NS	***
Tukey’s Multiple Range Test ^‡^															
Irrigation															
T0	2.062	0.692 a	1.370	17.10	10.78 a	9.48 ab	55	42	4	67.74 a		−5.93	32.60	33.15	100.28 a
T1	2.051	0.695 a	1.356	17.23	10.74 a	9.56 a	53	43	4	66.37 b		−5.63	32.98	33.47	99.69 ab
T2	2.009	0.673 b	1.335	17.06	10.49 b	9.35 b	54	42	4	66.44 b		−5.54	32.67	33.16	99.61 b
Rootstock															
*P. atlantica*	2.036	0.686 ab	1.350	17.16	10.62	9.43 b	50 b	46 a	4	66.95 ab		−5.39 a	32.86	33.31	99.27 b
*P. integerrima*	2.047	0.695 a	1.352	17.18	10.68	9.61 a	52 b	46 a	2	67.32 a		−6.34 b	32.51	33.13	101.02 a
*P. terebinthus*	2.038	0.679 b	1.359	17.05	10.71	9.36 b	60 a	35 b	5	66.29 b		−5.38 a	32.88	33.33	99.29 b
Irrigation × Rootstock															
T0 × *P. atlantica*	2.061	0.691 ab	1.369	17.16	10.71 abc	9.37 bc	50 b	46 a	4	66.68 bc		−5.79 ab	33.16	33.68	99.87 ab
T1 × *P. atlantica*	2.029	0.688 ab	1.340	17.28	10.73 ab	9.41 abc	49 b	47 a	4	66.90 bc		−5.61 ab	32.79	33.27	99.69 ab
T2 × *P. atlantica*	2.018	0.678 ab	1.339	17.04	10.42 c	9.47 abc	51 b	44 a	5	67.26 b		−4.75 a	32.61	32.97	98.24 b
T0 × *P. integerrima*	2.062	0.702 a	1.360	17.15	10.91 a	9.67 ab	53 b	45 a	4	68.75 a		−6.59 b	32.56	33.23	101.43 a
T1 × *P. integerrima*	2.061	0.702 a	1.358	17.16	10.61 abc	9.74 a	51 b	46 a	4	66.49 c		−5.84 ab	32.64	33.16	100.15 a
T2 × *P. integerrima*	2.017	0.681 ab	1.336	17.24	10.54 bc	9.41 abc	51 b	47 a	3	66.74 bc		−6.58 b	32.34	33.01	101.48 a
T0 × *P. terebinthus*	2.062	0.682 ab	1.379	16.99	10.75 ab	9.37 bc	62 a	34 b	4	67.79 ab		−5.41 ab	32.07	32.53	99.52 ab
T1 × *P. terebinthus*	2.062	0.693 ab	1.368	17.25	10.88 a	9.54 ab	60 a	36 b	4	65.74 cd		−5.44 ab	33.51	33.96	99.23 ab
T2 × *P. terebinthus*	1.990	0.661 b	1.329	16.91	10.51 bc	9.17 c	59 a	36 b	5	65.34 d		−5.29 ab	33.07	33.51	99.11 ab

^Ϯ^ NS: not significant at *p* < 0.05; * and ***: significant at *p* < 0.05 and 0.001, respectively. ^‡^ Values (mean of 3 replications) followed by the same letter, within the same column and factor, were not significantly different (*p* < 0.05), Tukey’s least significant difference test. **^§^** Others’ means unpeeled, broken shell, dark color, etc. L, a*, b*: CIEL*a*b* color coordinates.

## Supplementary Materials

The following are available online at https://www.mdpi.com/2304-8158/9/2/158/s1. Table S1: Identification and sensory descriptors of volatile compounds on pistachios affected by regulated deficit irrigation and rootstock.
