# Supplementary material for: Volatile, Sensory and Functional Properties of HydroSOS Pistachios
Source: Foods. 2020 Feb 6;9(2):158. doi: 10.3390/foods9020158 (PMC7074072; doi:10.3390/foods9020158)
Supplement: Supplementary file 1 [file foods-09-00158-s001.pdf]

Table S1. Identification and sensory descriptors of volatile compounds on pistachios affected by regulated deficit irrigation and rootstock

#	Compound	Rt (min)	KI (exp.)	KI (lit.)	Sensory descriptor
1	Acetic acid	2.21	603	605	Vinegar
2	Ethyl acetate	2.44	601	612	Pineapple; Anise
3	Pentanone	3.52	702	705	Fruity; Cheese; Chocolate
4	1-Methyl-1H-pyrrole	4.38	748	743	Woody; Smoky; Herbal
5	1-Pentanol	5.03	774	765	Sweet; Vanilla; Fusel
6	(Z)-3-Octene	5.56	792	790	nf
7	Hexanal	5.85	801	800	Fatty; Green
8	2-Octene	6.26	818	815	nf
9	1-Hexanol	7.95	874	868	Green; Herbaceous; Woody
10	(E)-4-Nonene	8.51	890	890	nf
11	(Z)-4-Nonene	8.64	893	893	nf
12	Nonane	8.90	900	900	Gasoline
13	α-Pinene	9.99	938	937	Woody
14	2-Pentanol	10.30	948	950	Oily; Green
15	1-Decene	11.21	975	981	nf
16	Sabinene	11.38	980	975	Woody; Pine; Spicy
17	3-Decene	11.71	989	988	nf
18	β-Myrcene	11.84	992	991	Fruity; Herbaceous; Sweet
19	Decane	12.15	1000	1000	Alkane
20	3-Carene	12.48	1012	1011	Lemon
21	Limonene	13.06	1032	1031	Citrus; Sweet
22	(E)-3-Hexenol	13.29	1040	1038	Green
23	2-Octen-1-ol	13.86	1057	1061	Citrus; Fruity; Green; Vegetable
24	2-Methyl-decane	14.19	1068	1064	nf
25	Terpinolene	14.95	1090	1089	Plastic
26	Undecane	15.31	1100	1100	Alkane
27	2-Nonen-1-ol	15.43	1106	1105	Melon; Waxy
28	1-Nonanol	17.39	1180	1176	Citrus; Rose
29	Dodecane	18.00	1201	1200	Alkane
30	Decanal	18.11	1205	1206	Floral; Citrus; Sweet; Waxy
31	Tridecane	19.48	1301	1300	Alkane

Rt = Retention time; KI (exp.)= Kovat's index experimental; KI (lit.) = Kovat's index literature; nf = not found.
